# Ultrasonographic Features, Nodule Size, Capsular Invasion, and Lymph Node Metastasis of Solitary Papillary Carcinoma of Thyroid Isthmus

**DOI:** 10.3389/fonc.2020.558363

**Published:** 2020-09-29

**Authors:** Honghao Luo, Feng Yan, Lin Lan, Buyun Ma, Haina Zhao, Yushuang He, Yulan Peng

**Affiliations:** Department of Ultrasound Medicine, West China Hospital of Sichuan University, Chengdu, China

**Keywords:** thyroid isthmus nodule, papillary thyroid carcinoma, ultrasound, lymph node metastasis, capsular invasion

## Abstract

**Objective:** This retrospective study aimed to analyze the ultrasound (US) imaging features of solitary papillary thyroid carcinoma (PTC) located in the isthmus and to assess the risk factors for lymph node metastasis (LNM) and tumor capsular invasion.

**Methods:** We included a total of 135 patients with solitary PTC located in the isthmus. All the cases underwent US, total thyroidectomy, and prophylactic central lymph node dissection. Patients' demographic and thyroid isthmus nodules' US characteristics, as well as risk factors associated with LNM and tumor capsular invasion, were analyzed.

**Results:** It was revealed that the occurrence of LNM was higher in male patients than in female patients (*P* < 0.001). As risk factors, the size of PTC in the isthmus was found to be associated with LNM and tumor capsular invasion (*P* = 0.005 and 0.000, respectively). The area under the receiver operating characteristic curve (AUC) of the size of the isthmus PTC was 0.64 [95% confidence interval (CI) = 0.55–0.72], indicating a probability for LNM. The AUC value for tumor capsular invasion was 0.77 (95% CI: 0.68–0.83). When the threshold was set to 1.1 cm, the larger size indicated that there was a probability of occurrence of LNM with sensitivity and specificity of 47.4 and 73.7%, respectively. When the threshold was set to 0.7 cm, the larger size indicated that there was potentially a tumor capsular invasion, with sensitivity and specificity of 80.6 and 56.3%, respectively. Wider-than-tall nodules were found to be significantly different from those in LNM and tumor capsular invasion (*P* = 0.038 and 0.030, respectively). There were significant differences in tumor capsular invasion in extrathyroidal extension (ETE) compared with smooth or ill-defined and lobulated or irregular nodules (*P* = 0.017).

**Conclusions:** This study showed that the incidence of LNM in male patients was higher than that in female ones. When a US image shows a thyroid isthmus nodule with a wider-than-tall shape, LNM and tumor capsular invasion were likely to occur. When a US image shows a thyroid isthmus nodule with an ETE, tumor capsular invasion was likely to occur. ETE and wider-than-tall may be indicators of FNA under US guidance, even though the size of thyroid isthmus nodule may be <1 cm.

## Introduction

The most frequent endocrine malignancy is thyroid cancer, and the most common histological subtype of thyroid cancer is papillary thyroid cancer (PTC), accounting for about 80% of all cases with thyroid cancer. PTC often occurs in patients 20–55 years old, especially in women (1–4). Although the 10-year survival rate of PTC is high, it can reach about 93%, whereas some special locations of PTC have shown certain invasive behavior, and about 30–90% of PTCs may have clinical or occult cervical lymph node metastasis (LNM) (5). PTC can occur anywhere in the thyroid, including the lateral lobe and isthmus; PTC in the isthmus is less than that in the lateral lobe, and PTC located in the isthmus is associated with incidence of 3–9.2% (3, 6–8). A number of scholars have studied PTC location in the isthmus; because the isthmus is located directly in front of the trachea, it covers the second to fourth tracheal rings, and it is covered by the strap muscles, fascia, and skin in the middle of the neck (8). Studies have shown that PTC is located in the isthmus with a higher rate of LNM, tumor capsular invasion, and extrathyroidal extension (ETE) (9–11).

At present, high-resolution ultrasound is the standard imaging method for thyroid nodule detection and diagnosis. Ultrasound-guided fine-needle aspiration (FNA) can determine the benignancy or malignancy of thyroid nodule. Therefore, a reliable, noninvasive method is required to determine which thyroid nodules can be diagnosed by FNA. In May 2017, the American College of Radiology (ACR) launched a classification system—Thyroid Imaging Reporting and Data System (TI-RADS), in which all thyroid nodules were divided into five categories: 1, 2, 3, 4, and 5 (12). However, the LNM-based patterns for PTC in the isthmus have not been fully elucidated.

The present study aimed to investigate the relationship between tumor capsular invasion and LNM associated with PTC in the isthmus. In addition, we assessed the frequency and pattern of LNM associated with PTC in the isthmus.

## Materials and Methods

This retrospective study was approved by the Ethics Committee of the West China Hospital, Sichuan University (Chengdu, China), and was granted a waiver of written informed consent for use of data.

### Patient Selection

**Figure 1:**
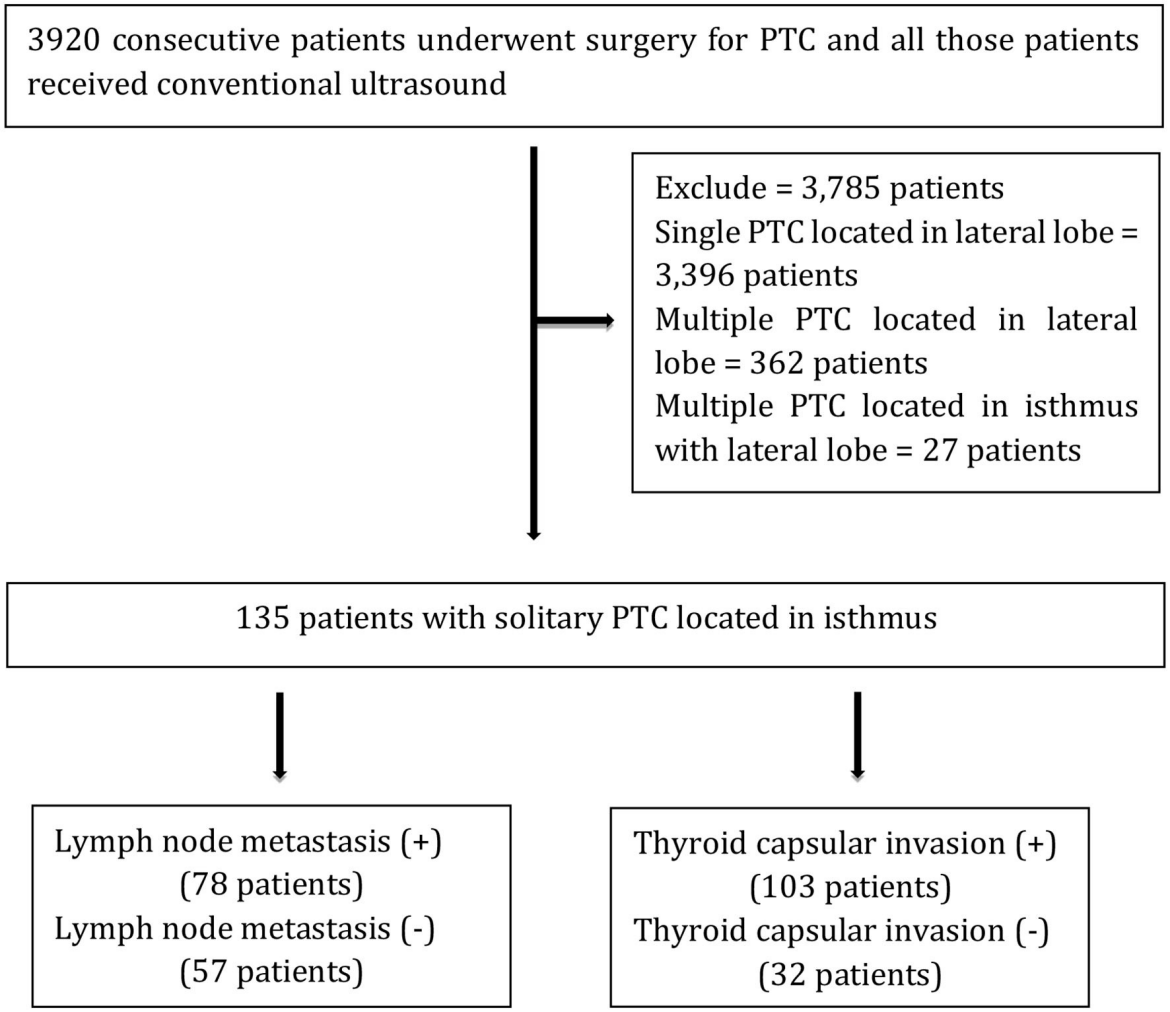
Flowchart of the study. +, pathological LN metastasis and capsular invasion; –, pathological no LN metastasis and capsular invasion.

From September 2016 to September 2018, the records of 3,920 consecutive patients who underwent surgery for PTC and received conventional ultrasound were extracted from a database at the Department of Ultrasound Medicine, West China Hospital, Sichuan University. Additionally, 27 patients with PTC located in the isthmus with the lateral lobe, 3,396 patients with single PTC located in the lateral lobe, and 362 patients with multiple PTC located in the lateral lobe were excluded. A total of 135 patients with solitary PTC located in the isthmus were included in the present study, and those were divided into two groups according to whether there was LNM and tumor capsular invasion as described in Figure 1. Moreover, 135 patients with solitary PTC located in the isthmus were involved in our study (women, 102; mean age, 42.0 ± 12.4 years old; range, 17–68 years old; men, 33; mean age, 42.1 ± 11.2 years old; range, 16–69 years old), and all patients underwent total thyroidectomy and bilateral central neck lymph node (LN) dissection; if necessary, cervical lateral LN dissection should be performed simultaneously. In the current research, all cases were diagnosed as PTC, and tumor capsular invasion and LNM were detected according to histopathological findings obtained from surgery.

### Ultrasound Examination and Image Analysis

Ultrasound images of thyroid nodule and cervical LNM were captured using Philips iU22 ultrasound system (Philips Healthcare, Eindhoven, the Netherlands) equipped with a 5- to 12-MHz linear-array transducer. Two sonographers (Buyun Ma and Honghao Luo, with 22 and 7 years of clinical experience in thyroid ultrasound) retrospectively analyzed ultrasound images and reached consensus. ACR TI-RADS was employed for standard evaluation of thyroid nodules, including composition (cystic or almost completely cystic, spongiform, mixed cystic and solid, solid, or almost completely solid), echogenicity (anechoic, hyperechoic or isoechoic, hypoechoic, very hypoechoic), shape (wider-than-tall, taller-than-wide), margin (smooth, ill-defined, lobulated or irregular, ETE), and echogenic foci [none or large comet-tail artifacts, macrocalcifications, peripheral (rim) calcification, punctate echogenic foci]. The following points were added from all categories to determine TI-RADS level as follows: 0 point (TR1, benign, no FNA), 2 points (TR2, not suspicious, no FNA), 3 points (TR3, mildly suspicious, FNA if ≥2.5 cm, follow if ≥1.5 cm), 4–6 points (TR4, moderately suspicious, FNA if ≥1.5 cm, follow if ≥1 cm), 7 points or more (TR5, highly suspicious, FNA if ≥1 cm, follow if ≥0.5 cm). The reviewers also assessed the ultrasound characteristics of cervical LN, and abnormal findings were suggestive of cervical LNM, including globular shape, loss of the normal echogenic hilum, presence of peripheral rather than hilar flow, heterogeneity with cystic components, and punctate echogenic foci that may represent microcalcifications and local hypoechoic in LN.

### Surgery and Pathological Diagnosis

In our hospital, all the patients with solitary PTC located in the isthmus underwent total thyroidectomy, and prophylactic central LN dissection was routinely performed. If ultrasound examination revealed abnormal LN located in the cervical lateral region, ultrasound-guided FNA was found highly essential, and it could indicate whether the LN was metastasis or not; if there was a cervical lateral LNM on an intraoperative frozen section, cervical lateral LN dissection should be simultaneously performed. All cases had been confirmed by postoperative pathological diagnosis.

### Statistical Analysis

The thyroid nodule was classified according to five categories on the basis of ACR TI-RADS. We used either the χ^2^ test or the Fisher exact test for comparing differences between categorical variables, and continuous variables were compared by the independent-samples *t*-test; for this purpose, demographic characteristics, thyroid nodule size, ultrasound manifestations, tumor capsular invasion, and cervical LNM were analyzed. *P* < 0.05 was considered statistically significant. The receiver operating characteristic (ROC) curves were plotted to find out the threshold of thyroid nodule size in the isthmus and to indicate the occurrence of LNM and tumor capsular invasion. The values of area under the ROC curve (AUC) and 95% confidence interval (CI) were calculated. Software 22.0 software (IBM, Armonk, NY, USA) was used to perform statistical analyses.

## Results

The size of solitary PTC located in the isthmus in the 135 cases ranged between 0.3 and 3.4 cm (average, 1.1 ± 0.6 cm). Nodules <1 cm accounted for 50.4% (68 of 135), and those >1 cm accounted for 49.6% (67 of 135).

### The Patients' Clinical and Pathological Characteristics

**Figure 2:**
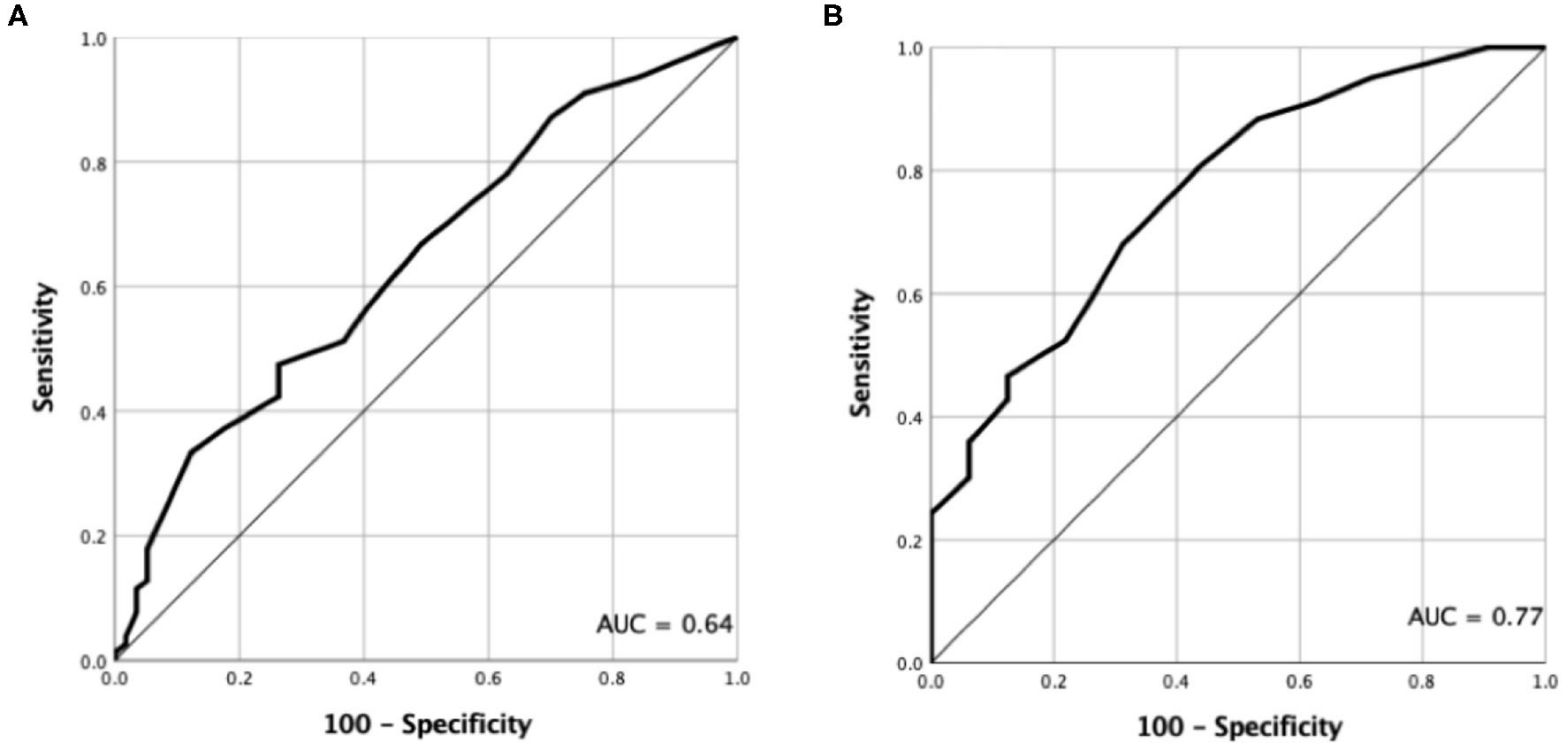
ROC curves for discerning between LN metastasis and tumor capsular invasion based on thyroid isthmus nodule size. **(A)** The AUC for lymph node metastasis was 0.64 (95% CI: 0.55–0.72). **(B)** The AUC for tumor capsular invasion was 0.77 (95% CI: 0.68–0.83).

Total thyroidectomy was performed on all the patients who were diagnosed with PTC. Depending on whether there is LNM and tumor capsular invasion, the patients' clinical and pathological characteristics are presented in Tables 1, 2. There were no significant differences in the patients' ages whether there was LNM and tumor capsular invasion (*P* = 0.607 and 0.863, respectively). The incidence of LNM was higher in males than that in females (81.8 vs. 50%, respectively, *P* < 0.001), and the incidence of tumor capsular invasion in males was similar to that in females (72.7 vs. 77.5%, respectively, *P* = 0.579). The diameter of thyroid nodule with LNM and tumor capsular invasion in the isthmus was larger than that in thyroid nodule with no LNM and tumor capsular invasion in the isthmus (*P* = 0.005 and 0.000, respectively). The AUC values for the size of thyroid nodule with LNM and tumor capsular invasion in the isthmus were 0.64 (95% CI: 0.55–0.72) and 0.77 (95% CI: 0.68–0.83), respectively. When the threshold was 1.1 cm, the size of thyroid nodule in the isthmus was >1.1 cm, which indicated that there was LNM, and the sensitivity and specificity were 47.4 and 73.7%, respectively. When the threshold was 0.7 cm, the size of thyroid nodule in the isthmus was >0.7 cm, which demonstrated that there was tumor capsular invasion, and the sensitivity and specificity were 80.6 and 56.3%, respectively (Figure 2).

**Table 1:** Lymph node metastasis in 135 patients.

	**Total**	**Positive**	**Negative**	***P***
No. of cases	135	78	57	
Age (y)	42.0 ± 12.1	41.5 ± 12.3	42.6 ± 11.9	0.607
**Sex**				0.001
Female	102	51	51	
Male	33	27	6	
Size of nodule (cm)	1.1 ± 0.6	1.2 ± 0.6	0.9 ± 0.5	0.005

**Table 2:** Thyroid capsular invasion in 135 patients.

	**Total**	**Positive**	**Negative**	***P***
No. of cases	135	103	32	
Age (y)	42.0 ± 12.1	42.2 ± 12.6	41.2 ± 10.3	0.863
**Sex**				0.579
Female	102	79	23	
Male	33	24	9	
Size of nodule (cm)	1.1 ± 0.6	1.2 ± 0.6	0.7 ± 0.4	0.000

### Analysis of Ultrasound Images

PTC located in the isthmus was solid (133/135, 98.5%), hypoechoic (135/135, 100%), wider-than-tall (100/135, 74.1%), lobulated or irregular (61/135, 45.2%), ETE (63/135, 46.7%), punctate echogenic foci (107/135, 79.3%), and TR5 (127/135, 94.1%).

According to the existence of LNM and tumor capsular invasion, the ultrasound imaging characteristics of PTC located in the isthmus were analyzed. The ultrasound imaging characteristics of the study subjects are summarized in Tables 3, 4.

**Table 3:** The ultrasound imaging characteristics with and without lymph node metastasis.

**Characteristic**	**Positive (78)**	**Negative (57)**	***P***
**Composition**			
Cystic or almost completely cystic	0	0	
Spongiform	0	0	
Mixed cystic and solid	2	0	0.508
Solid or almost completely solid	76	57	
**Echogenicity**			–
Anechoic	0	0	
Hyperechoic or isoechoic	0	0	
Hypoechoic	78	57	
Very hypoechoic	0	0	
**Shape**			0.038
Wider-than-tall	63	37	
Taller-than-wide	15	20	
**Margin**			0.140
Smooth or ill-defined	5	6	
Lobulated or irregular	31	30	
Extrathyroidal extension	42	21	
**Echogenic foci**			0.162
None or large comet-tail artifacts	8	11	
Macrocalcifications	2	4	
Peripheral (rim) calcifications	1	2	
Punctate echogenic foci	67	40	
**TI-RADS level**			0.282
TR4	3	5	
TR5	75	52	

**Table 4:** The ultrasound imaging characteristics with and without tumor capsular invasion.

**Characteristic**	**Positive (103)**	**Negative (32)**	***P***
**Composition**			
Cystic or almost completely cystic	0	0	
Spongiform	0	0	
Mixed cystic and solid	2	0	1.000
Solid or almost completely solid	101	32	
**Echogenicity**			–
Anechoic	0	0	
Hyperechoic or isoechoic	0	0	
Hypoechoic	103	32	
Very hypoechoic	0	0	
**Shape**			0.030
Wider-than-tall	81	19	
Taller-than-wide	22	13	
**Margin**			0.017
Smooth or ill-defined	8	3	
Lobulated or irregular	40	21	
Extrathyroidal extension	55	8	
**Echogenic foci**			0.721
None or large comet-tail artifacts	15	4	
Macrocalcifications	4	2	
Peripheral(rim) calcifications	3	0	
Punctate echogenic foci	81	26	
**TI-RADS level**			1.000
TR4	6	2	
TR5	97	30	

**Figure 3:**
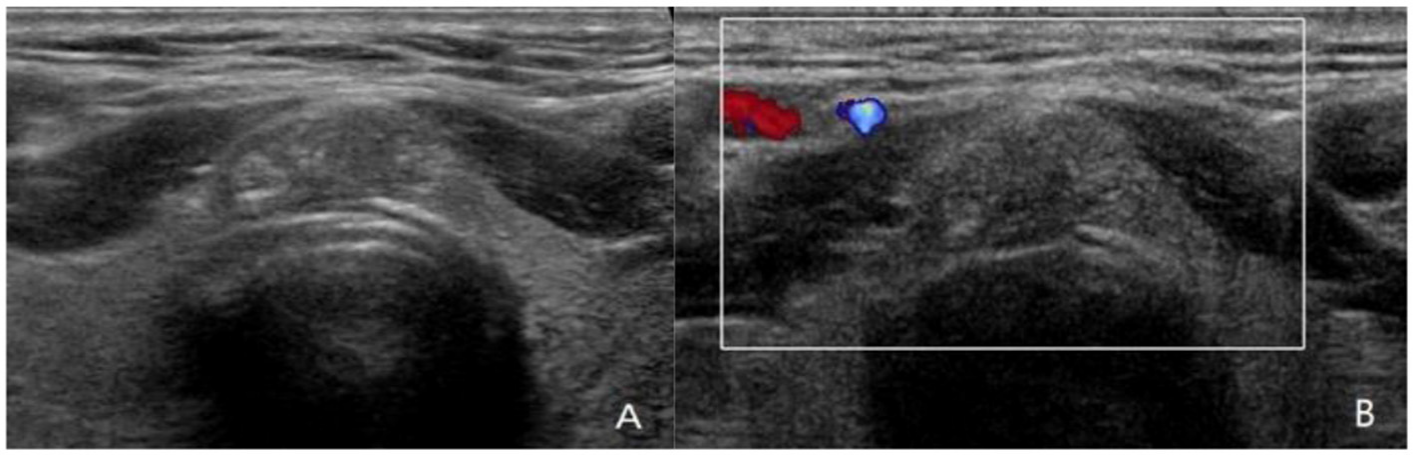
A 42-year-old man with papillary thyroid carcinoma (PTC) located in isthmus. **(A,B)** Ultrasound images show 1.3-cm solid nodule with hypoechoic, wider-than-tall, extrathyroidal extension (ETE), punctate echogenic foci, ACR TI-RADS 5. PTC was confirmed by fine-needle aspiration (FNA) cytologic examination before operation. The PTC in isthmus of thyroid was confirmed by postoperative pathology, with capsular invasion and bilateral paratracheal lymph node metastasis. Although ultrasound showed ETE, no abnormal lymph node was found.

Wider-than-tall was found to be significantly different from both LNM and tumor capsular invasion-positive in the analysis of ultrasound characteristics (*P* = 0.038 and 0.030, respectively). There were significant differences in tumor capsular invasion between ETE and smooth or ill-defined and lobulated or irregularly shaped tumor (*P* = 0.017). Composition, echogenicity, echogenic foci, and TI-RADS level were found insignificantly different from both LNM and tumor capsular invasion-positive (Figure 3).

### LNM Analysis

There were 78 of 135 cases (57.8%) who were confirmed to have LNM by postoperative pathology, including central LN (77/135, 57%; there were 69 cases with paratracheal LNM and 21 cases with pretracheal LNM) and lateral LN (6/135, 4.4%).

### The Relationship Between LNM and Tumor Capsular Invasion

According to the existence of tumor capsular invasion, there were 103 cases with tumor capsular invasion, of whom 64 (62.1%) cases had LNM, and 39 (37.9%) cases had no LNM; among 32 cases without tumor capsular invasion, 14 (43.8%) cases had LNM, whereas 18 (56.2%) cases had no LNM.

Although LNM mainly occurred in tumor capsular invasion than in nontumor capsular invasion, there was no significant difference between the two groups (*P* = 0.066).

## Discussion

The thyroid is divided into upper third, middle third, lower third, and the isthmus (11). The isthmus is located in front of the trachea; it is connected with bilateral lobes of the thyroid, with a thickness of 2–6 mm and small-volume thyroid parenchyma (13). The lymphatic drainage of the thyroid gland involves the lower deep cervical, prelaryngeal, pretracheal, and paratracheal nodes. The paratracheal and lower deep cervical nodes receive lymphatic drainage from the isthmus and the inferior lateral lobes (14). A study performed by Song et al. (7) showed that PTC located in the isthmus is an independent risk factor for central LNM. Because of the special location of the isthmus and difference in drainage of LN, the surgical protocols for PTC in the isthmus are also different from PTC in the lateral lobe. Numerous scholars have concentrated on evaluating the efficacy of surgery for PTC in the isthmus, while they have scarcely studied LNM and tumor management, and involved a limited number of cases (13, 15, 16). Previous studies have shown that PTC located in the isthmus has a low incidence, whereas it is more prone to invade neighboring organizations. In the current study, we selected patients with solitary PTC located in the isthmus who underwent total thyroidectomy and preventive central LN dissection, and we found that the morbidity of PTC located in the isthmus of thyroid was 4.1% (162/3,920 cases), which is similar to that reported by previous studies (3, 6–8).

The present study showed that the morbidity of PTC in the isthmus is significantly higher in females than that in males, with a ratio of 3:1. Although the incidence of PTC in the isthmus in males is low, it is more likely to have LNM (*P* = 0.001). There was no association among LNM, tumor capsular invasion, and patients' age (*P* = 0.607and 0.863, respectively).

Although tumor size is a factor in LNM and tumor capsular invasion, a number of scholars (8) have pointed out that there was no statistically significant difference between LNM and tumor size <10 and >10 mm, suggesting that the isthmus location could be a risk factor for central LN involvement, regardless of tumor size. On the contrary, the present study showed that the size of PTC in the isthmus with LNM is significantly larger than that without LNM (*P* = 0.005). Wang et al. (17) analyzed factors related to central LNM in 73 patients with solitary PTC in the isthmus and found that tumor size >0.7 cm was associated with central LNM and tumor size >0.6 cm was significantly associated with paratracheal LNM. Our findings revealed that tumor size >1.1 cm is significantly associated with LNM (AUC = 0.64, sensitivity = 47.4%, and specificity = 73.7%). Meanwhile, the present research demonstrated that the size of PTC in the isthmus with tumor capsular invasion was significantly larger than that without tumor capsular invasion (*P* = 0.000). Our study uncovered that tumor size >0.7 cm is significantly correlated with tumor capsular invasion (AUC = 0.77, sensitivity = 80.6%, and specificity = 56.3%). In our study, 50.4% (68/135) of cases had PTC in the isthmus with size of ≤1 cm, and in such cases, the rate of LNM and tumor capsular invasion reached 50% (34/68) and 64.7% (44/68), respectively. Therefore, we can infer that when the size of PTC in the isthmus is small, the tumor capsular invasion can occur. When PTC in the isthmus reaches a certain size, the LNM may occur.

High-resolution ultrasound has been widely used in thyroid examination. Besides, PTC can be predicted from certain suspicious ultrasound features of thyroid nodules. When ultrasound displayed that thyroid nodule has hypoechoic or very hypoechoic, solid, taller-than-wide, ETE, punctate echogenic foci, the existence of a malignant tumor is more probable (16). However, we found that PTC located in the isthmus had more frequently the characteristics of wider-than-tall shape when they had LNM and tumor capsular invasion (*P* = 0.038 and 0.030, respectively), and ETE was more prone to have tumor capsular invasion than lobulated or irregularly shaped tumor (*P* = 0.017). These ultrasound characteristics seem to be due to the narrow space in the isthmus of the thyroid, which limits the longitudinal growth of PTC, while it does not hinder the transverse growth. The larger the PTC located in isthmus, the more likely the wider-than-tall shape.

The relationship between PTC location and cervical LNM remains controversial (11, 15–18). In (3) study, 181 PTCs in the isthmus were included, and central LNM occurred in 40.3% of cases. Song et al. (7) showed that the involvement of central LNM of PTC in the isthmus reached 71.1% and demonstrated that PTC located in the isthmus was an individual risk factor for central LNM, which was higher than the rate of 57.8% presented in the current study. Their cases included multifocal PTC located in both the isthmus and lateral lobes, whereas in our study, only PTC in the isthmus was involved, justifying this discrepancy. PTC located in the isthmus was prone to occult LNM, because of air disturbance from the trachea, and preoperative ultrasound revealed that abnormal LN in the central region was more difficult than that in the lateral region; thus, the diagnostic rate of ultrasound for central LNM was low (19). Therefore, further ultrasound assessment should be undertaken on the suspicious malignant nodules that originated from the isthmus, especially for the cervical LN.

The thyroid lymphatic drainage system is parallel to the thyroid venous drainage system (20), which are mainly divided into pretracheal, prelaryngeal, and paratracheal lymph nodes, especially superiorly to the prelaryngeal LN (21). As shown in the current research, the LNM rate was 57.8%, and paratracheal LNM was the most common type (88.5%, 69/78), followed by pretracheal LNM (26.9%, 21/78), and the main reason is that PTC in the isthmus lies directly anterior to the trachea, overlying the tracheal rings in the middle of the neck (22). The rate of lateral LNM in the isthmus PTC was similar to that in the literature (9.4%) (3). We found similar rates of central and lateral LNM in presence or absence of tumor thyroid capsular.

We also found that PTC originating in the isthmus more frequently had a pathologic ETE, and among various prognostic factors, ETE was found to be associated with LNM, distant metastasis, local recurrence, and cancer-related mortality (23–25). The reason could be related to the particular anatomical location of the isthmus, in which larger isthmus tumors are more prone to invade the thyroid capsular, and capsular invasion cases are more susceptible to invade neighboring organizations. In the present study, it was revealed that although the incidence of LNM was higher when the thyroid capsular was invaded, however, there was no significant difference between LNM and with/without tumor capsular invasion.

In the current study, we included 135 patients with solitary PTC located in the isthmus who underwent total thyroidectomy and central LN dissection. This retrospective study revealed the risk factors for LNM from solitary PTC located in the isthmus, in addition to the relationship between LNM and tumor capsular invasion and tumor size. Because PTC in the isthmus is located in the middle of the trachea, the tumors typically spread to paratracheal LN and pretracheal LN. Our findings suggest that PTC in the isthmus should be dissected with bilateral paratracheal LN and pretracheal LN, while the effect of prevention of central LN in the isthmus PTC needs further research and long-term follow-up.

A guideline (26) prohibited performing FNA for nodule size <1 cm, and it recommended that if the nodule was limited to the thyroid, its ultrasound characteristics need not be considered. Other guidelines (27–29) suggest that if the cytological diagnosis is confirmed to be PTC and the nodule is <1 cm, it is more appropriate for effective active surveillance. A number of studies reported that thyroid cancer is more invasive and is associated with poor prognosis when it is located in the isthmus (3, 14, 30–32). Previous literature reported that if PTC is located in the isthmus, capsular invasion and ETE are not associated with tumor size, and these findings were also frequently observed in the papillary thyroid microcarcinoma located in the isthmus (8). Some studies have confirmed that the frequency of LNM in PTC located in the isthmus is higher than the PTC located in the lateral lobes (7, 33). In the current study, we found that 50% (34/68) of the isthmus PTC may have LNM, and 64.7% (44/68) of the isthmus PTC may have tumor capsular invasion. Therefore, we believe that FNA should be actively carried out for nodules with size of <1 cm in the isthmus in order to carry out the next clinical intervention.

The learning-based methods focused on automatic learning from ultrasound images to conclude a final evaluation. These methods construct highly nonlinear mapping from featured spaces to the final evaluation (34). The featured spaces are often redundant in features, because extracted features from ultrasound images make it easy to be affected by noise and other tissues. In contrast, our method contributes to establish the relationship between the direct clinical indicators (e.g., size of nodule) and final evaluation (35).

Our present study had several limitations. First, the sample size of the single isthmus PTC was relatively small, because this location was rare; we also excluded multiple PTCs located in the isthmus and in the lobe, which may cause a selection bias. Second, because of small sample size, statistical analysis was not performed for different regions of cervical LNM. Third, our study did not statistically analyze the positive results of the ultrasound suspicious for abnormal LN, because the diagnosis of LNM is more difficult on preoperative ultrasound examination, in which it is mainly caused by air interference of trachea and some potential LNM. Lastly, our study sample was the single pathological type and confined to solitary PTC located in the isthmus, and further studies should be conducted to evaluate the solitary PTC located in the isthmus and lateral lobe in LNM and tumor capsular invasion.

## Conclusions

The present results demonstrated that the incidence of LNM in male patients with PTC in the isthmus was higher than that in female patients. The aspects of nodule size and wider-than-tall shape were found to be risk factors for LNM and tumor capsular invasion, and the ETE was the risk factor for tumor capsular invasion. We also found that there was no association between the occurrence of tumor capsular invasion and LNM, and LNM was mostly in the central LN, especially the paratracheal LN. Therefore, the PTC in the isthmus should be carefully evaluated by ultrasound. ETE and wider-than-tall shape may be indicators of FNA under ultrasound guidance, even though the size of thyroid nodule may be <1 cm.

## Data Availability Statement

The raw data supporting the conclusions of this article will be made available by the authors, without undue reservation.

## Ethics Statement

This retrospective study was approved by the Ethics Committee of the West China Hospital of Sichuan University and was granted a waiver of written informed consent for use of data.

## Author Contributions

HL and FY participated in the study design, and contributed to the data collection, and draft the manuscript. LL, YH, BM, HZ, and YP made important contributions in collecting and analyzing data, and in revising the content. All authors read and approved the final manuscript.

## Conflict of Interest

The authors declare that the research was conducted in the absence of any commercial or financial relationships that could be construed as a potential conflict of interest.

## References

[1] Siegel RL Miller KD Jemal A Cancer statistics, 2017. CA Cancer J Clin. (2017) 67:7–30. 10.3322/caac.21387 28055103

[2] McLeod DS Sawka AM Cooper DS. Controversies in primary treatment of low-risk papillary thyroid cancer. Lancet. (2013) 381:1045–57. 10.1016/S0140-6736(12)62205-3 23668555

[3] Lee YS Jeong JJ Nam KH Chang HS Park CS. Papillary carcinoma located in the thyroid isthmus. World J Surg. (2010) 34:36–9. 10.1007/s00268-009-0298-6 20020291

[4] Sugenoya A Shingu K Kobayashi S Masuda H Takahashi S Shimizu T. Surgical strategies for differentiated carcinoma of the thyroid isthmus. Head Neck. (1993) 15:158–60. 10.1002/hed.2880150212 8440615

[5] Ito Y Miyauchi A Kihara M Higashiyama T Kobayashi K Miya A. Patient age is significantly related to the progression of papillary microcarcinoma of the thyroid under observation. Thyroid. (2014) 24:27–34. 10.1089/thy.2013.0367 24001104 PMC3887422

[6] Iyer NG Shaha AR. Management of thyroid nodules and surgery for differentiated thyroid cancer. Clin Oncol (R Coll Radiol). (2010) 22:405–12. 10.1016/j.clon.2010.03.009 20381323 PMC4806860

[7] Song CM Lee DW Ji YB Jeong JH Park JH Tae K. Frequency and pattern of central lymph node metastasis in papillary carcinoma of the thyroid isthmus. Head Neck. (2016) 38:E412–E6. 10.1002/hed.24009 25581039

[8] Karazas T Charitoudis G Vasileiadis D Kapetanakis S Vasileiadis I. Surgical treatment for dominant malignant nodules of the isthmus of the thyroid gland: a case control study. Int J Surg. (2015) 18:64–8. 10.1016/j.ijsu.2015.04.039 25900600

[9] Zhang L Wei WJ Ji QH Zhu YX Wang ZY Wang Y. Risk factors for neck nodal metastasis in papillary thyroid microcarcinoma: a study of 1066 patients. J Clin Endocrinol Metab. (2012) 97:1250–7. 10.1210/jc.2011-1546 22319042

[10] Pontieri G Urselli F Peschi L Liccardi A Ruggiero AR Vergara E. Is the isthmus location an additional risk factor for indeterminate thyroid nodules? Case report and review of the literature. Front Endocrinol (Lausanne). (2018) 9:750. 10.3389/fendo.2018.00750 30631304 PMC6315157

[11] Caron NR Clark OH. Papillary thyroid cancer: surgical management of lymph node metastases. Curr Options Oncol. (2005) 6:311–22. 10.1007/s11864-005-0035-9 15967084

[12] Tessler FN Middleton WD Grant EG Hoang JK Berland LL Teefey SA. ACR thyroid imaging, reporting and data system (TI-RADS): white paper of the ACR TI-RADS committee. J Am Coll Radiol. (2017) 14:587–95. 10.1016/j.jacr.2017.01.046 28372962

[13] Nixon IJ Palmer FL Whitcher MM Shaha AR Shah JP Patel SG. Thyroid isthmusectomy for well-differentiated thyroid cancer. Ann Surg Oncol. (2011) 18:767–70. 10.1245/s10434-010-1358-8 20882418

[14] Chai YJ Kim SJ Choi JY Koo do H Lee KE Youn YK. Papillary thyroid carcinoma located in the isthmus or upper third is associated with Delphian lymph node metastasis. World J Surg. (2014) 38:1306–11. 10.1007/s00268-013-2406-x 24366273

[15] Goldfarb M Rodgers SS Lew JI Appropriate surgical procedure for dominant thyroid nodules of the isthmus 1cm or lager. Arch Surg. (2012) 147:881–4. 10.1001/archsurg.2012.728 22987188

[16] Skilbeck C Leslie A Simo R. Thyroid isthmusectomy: a critical appraisal. J Laryngol Otol. (2007) 121:986–9. 10.1017/S0022215106005238 17156579

[17] Wang J Sun H Gao L Xie L Cai X. Evaluation of thyroid isthmusectomy as a potential treatment for papillary thyroid carcinoma limited to the isthmus: a clinical study of 73 patients. Head Neck. (2016) 38:E1510–4. 10.1002/hed.24270 26558441

[18] Kwak JY Kim EK Kim MJ Son EJ Chung WY Park CS. Papillary microcarcinoma of the thyroid: predicting factors of lateral neck node metastasis. Ann Surg Oncol. (2009) 16:1348–55. 10.1245/s10434-009-0384-x 19224278

[19] Park KN Kang KY Hong HS Jeong HS Lee SW. Predictive value of estimated tumor volume measured by ultrasonography for occult central lymph node metastasis in papillary thyroid carcinoma. Ultrasound Med Biol. (2015) 41:2849–54. 10.1016/j.ultrasmedbio.2015.02.018 26292989

[20] Mohebati A Shaha AR. Anatomy of thyroid and parathyroid glands and neurovascular relations. Clin Anat. (2012) 25:19–31. 10.1002/ca.21220 21800365

[21] Nakayama M Seino Y Okamoto M Mikami T Okamoto T Miyamoto S. Clinical significance of positive Delphian node in supracricoid laryngectomy with cricohyoidoepiglottopexy. Jpn J Clin Oncol. (2011) 41:987–91. 10.1093/jjco/hyr091 21715365

[22] Hahn SY Han BK Ko EY Shin JH Ko ES. Ultrasound findings of papillary thyroid carcinoma originating in the isthmus: comparison with lobe-originating papillary thyroid carcinoma. Am J Roentgenol. (2014) 203:637–42. 10.2214/AJR.13.10746 25148169

[23] Marqusee E Benson CB Frates MC Doubilet PM Larsen PR Cibas ES. Usefulness of ultrasonography in the management of nodular thyroid disease. Ann Intern Med. (2000) 133:696–700. 10.7326/0003-4819-133-9-200011070-00011 11074902

[24] Andersen PE Kinsella J Loree TR Shaha AR Shah JP. Differentiated carcinoma of the thyroid with extrathyroidal extension. Am J Surg. (1995) 170:467–70. 10.1016/S0002-9610(99)80331-6 7485734

[25] McCaffrey TV Bergstralh EJ Hay ID. Locally invasive papillary thyroid carcinoma: 1940-1990. Head Neck. (1994) 16:165–72. 10.1002/hed.2880160211 8021137

[26] Haugen BR Alexander EK Bible KC Doherty GM Mandel SJ Nikiforov YE. 2015 American thyroid association management guidelines for adult patients with thyroid nodules and differentiated thyroid cancer: the American Thyroid Association guidelines task force on thyroid nodules and differentiated thyroid cancer. Thyroid. (2016) 26:1–133. 10.1089/thy.2015.0020 26462967 PMC4739132

[27] Shin JH Baek JH Chung J Ha EJ Kim JH Lee YH. Ultrasonography diagnosis and imaging-based management of thyroid nodules: revised Korean Society of Thyroid Radiology consensus statement and recommendations. Korean J Radiol. (2016) 17:370–95. 10.3348/kjr.2016.17.3.370 27134526 PMC4842857

[28] Brito JP Ito Y Miyauchi A Tuttle RM. A clinical framework to facilitate risk stratification when considering an active surveillance alternative to immediate biopsy and surgery in papillary microcarcinoma. Thyroid. (2016) 26:144–9. 10.1089/thy.2015.0178 26414743 PMC4842944

[29] Ha SM Baek JH Na DG Suh CH Chung SR Choi YJ. Diagnostic performance of practice guidelines for thyroid nodules: thyroid nodule size versus biopsy rates. Radiology. (2019) 291:92–9. 10.1148/radiol.2019181723 30777805

[30] Lee YC Na SY Chung H Kim SI Eun YG. Clinicopathologic characteristics and pattern of central lymph node metastasis in papillary thyroid cancer located in the isthmus. Laryngoscope. (2016) 126:2419–21. 10.1002/lary.25926 27098428

[31] Lim ST Jeon YW Suh YJ. Correlation between surgical extent and prognosis in node-negative, early-stage papillary thyroid carcinoma originating in the isthmus. World J Surg. (2016) 40:344–9. 10.1007/s00268-015-3259-2 26446448

[32] Lei J Zhu J Li Z Gong R Wei T. Surgical procedures for papillary thyroid carcinoma located in the thyroid isthmus: an intention-to-treat analysis. Onco Targets Ther. (2016) 9:5209–5216. 10.2147/OTT.S106837 27578987 PMC5001660

[33] Xiang D Xie L Xu Y Li Z Hong Y Wang P. Papillary thyroid microcarcinomas located at the middle part of the middle third of the thyroid gland correlates with the presence of neck metastasis. Surgery. (2015) 157:526–33. 10.1016/j.surg.2014.10.020 25433730

[34] Chi J Walia E Babyn P Wang J Groot G Eramian M. Thyroid nodule classification in ultrasound images by fine-tuning deep convolutional neural network. J Digit Imaging. (2017) 30:477–86. 10.1007/s10278-017-9997-y 28695342 PMC5537102

[35] Ma J Wu F Zhu J Xu D Kong D. A pre-trained convolutional neural network based method for thyroid nodule diagnosis. Ultrasonics. (2017) 73:221–30. 10.1016/j.ultras.2016.09.011 27668999

